# Regulation of Atherosclerosis by Toll-Like Receptor 4 Induced by Serum Amyloid 1: A Systematic In Vitro Study

**DOI:** 10.1155/2022/4887593

**Published:** 2022-09-15

**Authors:** Jinhui Chen, Gang Liu, Yan Hong, Jing Han, Zhe Yang, Yanping Yang, Hong Li, Shumin Wang, Lili Jue, Qi Wang

**Affiliations:** ^1^Department of Histology and Embryology, School of Basic Medicine, Guizhou Medical University, Guiyang, Guizhou 550025, China; ^2^Guizhou Provincial Key Laboratory of Pathogenesis and Drug Research on Common Chronic Diseases, Guizhou Medical University, Guiyang, Guizhou 550025, China; ^3^Guizhou Provincial Engineering Technology Research Center for Chemical Drug R&D, Guizhou Medical University, Guiyang, Guizhou 550025, China; ^4^Department of Human Anatomy, School of Medicine, Zhengzhou University of Industrial Technology, Zhengzhou, Henan 450000, China

## Abstract

The objective of this study was to investigate the effects of serum amyloid 1 (SAA1) on activation of endothelial cells, formation of foam cells, platelet aggregation, and monocyte/platelet adhesion to endothelial cells. The effect of SAA1 on the inflammatory activation of endothelial cells was investigated by detecting the expression of inflammatory factors and adhesion molecules. The role of SAA1 in formation of foam cells was verified by detecting lipid deposition and expression of molecules related to the formation of foam cells. After platelets were stimulated by SAA1, the aggregation rate was evaluated to determine the effect of SAA1 on platelet aggregation. Monocytes/platelets were cocultured with human umbilical vein endothelial cells (HUVECs) pretreated with or without SAA1 to determine whether SAA1 affected monocyte/platelet adhesion to endothelial cells. By inhibiting toll-like receptor 4 (TLR4) function, we further identified the role of TLR4 signaling in SAA1-mediated endothelial inflammatory activation, foam-cell formation, and monocyte/platelet adhesion to HUVECs. SAA1 significantly increased the expression of adhesion molecules and inflammatory factors in HUVECs. Moreover, SAA1 also promoted lipid deposition and the expression of inflammatory factors and low-density lipoprotein receptor-1 (LOX-1) in THP-1-derived macrophages. In addition, SAA1 induced platelet aggregation and enhanced monocyte/platelet adhesion to HUVECs. However, the TLR4 antagonist significantly inhibited SAA1-induced endothelial cell activation, foam-cell formation, and monocyte/platelet adhesion to HUVECs and downregulated the expression of myeloid differentiation factor 88 (MyD88), phosphor-inhibitor of nuclear factor *κ*B kinase subunit *α*/*β* (P-IKK*α*/*β*), phospho-inhibitor of nuclear factor *κ*B subunit *α* (P-IKB*α*), and phosphorylation of nuclear transcription factor-*κ*B p65 (P-p65) in SAA1-induced HUVECs and THP-1 cells. Conclusively, it is speculated that SAA1 promotes atherosclerosis through enhancing endothelial cell activation, platelet aggregation, foam-cell formation, and monocyte/platelet adhesion to endothelial cells. These biological functions of SAA1 may depend on the activation of TLR4-related nuclear factor-kappa B (NF-*κ*B) signaling pathway.

## 1. Introduction

Cardiovascular disease is a serious threat to human health and is mainly related to hyperlipidemia, hypercholesterolemia, and chronic inflammation caused by abnormal metabolism [1, 2]. Atherosclerosis, the major pathological feature of cardiovascular disease, is caused by a series of pathological changes in cellular function, such as inflammatory activation of endothelial cells, lipid phagocytosis by macrophages, lipid internalization by vascular smooth muscle cells, and adhesion/aggregation of activated platelets that present in an alternate or overlapping manner, leading to vascular stenosis or blood flow blockage [3]. Currently, although tissue/organ ischemia and hypoxia induced by atherosclerosis can be treated by reducing the blood low-density lipoprotein (LDL) cholesterol level and inhibiting inflammatory responses, there are some drawbacks in these treatment strategies, such as low effectiveness and side effects [3]. Especially, in the development of atherosclerosis associated with metabolic syndrome, there are more unknown mechanisms to be explored because of intricate pathological cellular or molecular events. Elucidating these unknown mechanisms can not only help us understand the development of atherosclerosis but also provide a more theoretical basis for seeking more advanced therapeutic strategies.

Under the influence of risk factors such as hypercholesterolemia, metabolic syndrome, hypertension, inflammatory factors, and abnormal hemodynamics, endothelial cells undergo a series of functional changes, including high expression of adhesion molecules such as E-selectin (SELE), vascular cell adhesion molecule 1 (VCAM1), procoagulant factors (tissue factor/von Willebrand factor (VWF)), and chemoattractant protein (interleukin-8 (IL-8) and monocyte chemoattractant protein-1 (MCP-1)) and enhanced inflammatory signaling (NF-*κ*B signaling) activation, resulting in dysfunction or inflammatory activation of endothelial cells [4]. In inflammatory-activated endothelial cells, the highly expressed adhesion molecules facilitate the adhesion of leukocytes to endothelial cells, and the highly expressed inflammatory factors and chemokines can enhance the local vascular inflammatory response and increase the sensitivity of endothelial cells to systemic oxidized-low-density lipoprotein (ox-LDL) [5]. Platelet hyperreactivity and thrombosis are critical for dyslipidemia-induced atherosclerosis dependent on toll-like receptor 2- (TLR2-) mediated inflammatory signaling [6]. VWF released by activated endothelial cells binds to hypersensitive platelets via the glycoprotein IB-IX-V (GPIB-IX-V) complex to enhance local recruitment of leukocytes in blood vessels [7]. Moreover, the interaction between platelet P-selectin and endothelial P-selectin glycoprotein ligand-1 (PSGL-1) promotes platelets to roll over inflammatory-activated endothelial cells, thereby enhancing monocyte and neutrophil recruitment [8–10]. Monocytes recruited to damaged blood vessels will soon transdifferentiate into M1-type macrophages and secrete a large number of inflammatory factors to maintain continuous vascular inflammation. Meanwhile, these macrophages engulf lipids, leading to the formation of foam cells, which is also a hallmark of atherosclerosis [11, 12]. Therefore, elucidating the mechanisms that regulate endothelial cell activation, platelet hyperreactivity, and foam-cell formation is conducive to understanding the pathological process of atherogenesis.

SAA1, the main stress response protein in the host, is often highly expressed in acute or chronic stress such as infection and trauma. At present, it is widely believed that SAA1 is a special protein with the potential to promote inflammatory responses. SAA1 not only serves as one of the signs of injury or inflammatory response but also can enhance the secretion of inflammatory factors in immune cells, leading to further aggravation of injury or inflammation [13]. The functions of SAA1 mentioned above mainly depend on its binding to cell-surface receptors (formyl peptide receptor 2 (FPR2), TLR2, TLR4, and P2X7 purinergic receptor (P2RX7)) and activation of related inflammatory signaling (NF-*κ*B signaling) [14–16]. Recently, additional studies have proved that the upregulation of SAA1 is closely related to the occurrence and development of metabolic syndrome, obesity, and diabetes, all risk factors for atherosclerosis [17–20]. In particular, SAA1 has been shown to promote the formation of foam cells in vitro and atherosclerosis in vivo [21, 22]. However, there are no studies that have systematically investigated the role and possible mechanism of SAA1 in influencing the function of atherogenesis-related cells (platelets, endothelial cells, and foam cells).

A previous study has shown that SAA1 is an agonist of TLR4 [14], and SAA1 increased neuroinflammation through activating TLR4-related signaling in glial cells in a recent study [23]. In addition, TLR4 is a specialized receptor protein found in immune cells [24, 25] and is distributed on the surface of platelets, monocytes, and endothelial cells, and the activation of TLR4 signaling is also an important factor accelerating platelet aggregation, monocyte inflammatory phenotypic differentiation, and endothelial cell activation [26–28]. Here, through a series of in vitro experiments, we show that SAA1 plays a positive role in promoting platelet aggregation, inflammatory activation of endothelial cells, the formation of foam cells, and the adhesion of monocyte/platelet to endothelial cells and that such effects may be partly dependent on the activation of TLR4 signaling.

## 2. Materials and Methods

### 2.1. Cell Culture and Treatment

HUVEC lines (PNS-HC-65, Procell, Wuhan) were cultured using Dulbecco's Modified Eagle's Medium (DMEM, 8121308, Gibco, USA) supplemented with 10% fetal bovine serum (FBS, 10270106, Gibco, USA), 1% nonessential amino acids (PB180424, Procell, Wuhan), and 0.01 mg/ml insulin (PB180432, Procell, Wuhan) in a 5% CO_2_ incubator. When fused to 90%, the cells were passaged in a ratio of 1 : 3. HUVECs were treated by 10 *μ*g/ml SAA1 (300-53, PeproTech, USA) or SAA1+TAK-242 (1 *μ*g/ml, Invitrogen, USA) for 24 h, with the untreated HUVECs as the control. THP-1 human mononuclear cell lines (CL-0233, Procell, Wuhan) were cultured in RPMI 1640 medium (8121312, Gibco, USA) containing 10% FBS, and when fused to 90%, they were passaged. THP-1 cells were induced by 50 ng/ml phorbol-12-myristate-13-acetate (PMA, MKCC9510, Sigma, USA) for 12 h to differentiate into macrophages. They were, respectively, stimulated by SAA1 (10 *μ*g/ml), ox-LDL (50 *μ*g/ml), SAA1 (10 *μ*g/ml)+ox-LDL (50 *μ*g/ml), SAA1 (10 *μ*g/ml)+ox-LDL (50 *μ*g/ml)+TAK-242 (1 *μ*g/ml), or TAK-242 (1 *μ*g/ml) for 24 h, with the untreated macrophages as the control. The protein and messenger RNA (mRNA) of HUVECs or THP-1 cells were extracted for the following experiments.

### 2.2. Formation and Identification of Foam Cells

THP-1-differentiated macrophages were grown under various conditions and then fixed with Oil Red O fixative (G1262, Solarbio, Beijing) for 20 min. To observe the lipid deposition, the fixed cells were exposed to Oil Red O for 30 min, and the cellular images were captured by inverted phase contrast microscopy (Ti-u-dsri2, Nikon, Japan).

### 2.3. Adhesion of Monocytes to Endothelial Cells

THP-1 cells were incubated with 5 *μ*M of calcein AM (C8950, Solarbio, Beijing) for 15 min and then cocultured with HUVECs treated with SAA1 (10 *μ*g/ml) or with SAA1 (10 *μ*g/ml)+TAK-242 (1 *μ*g/ml) for 6 h, with the untreated HUVECs as the control. The cocultured cells were fixed with 4% paraformaldehyde (BL539A, Biosharp, Beijing) for 40 min, and the adhesive monocytes were observed by inverted fluorescence microscopy.

### 2.4. Preparation of Washed Platelets

The use of human blood samples was ratified by the Human Ethics Committee of Guizhou Medical University (No. 2021 (35)), and samples were provided by healthy, aspirin-free volunteers who gave informed written consent. After the fresh blood was mixed with 0.38% sodium citrate solution at a ratio of 9 : 1, the platelet-rich supernatant was collected and mixed with Tyrode buffer in equal volume and then centrifuged at 200 × *g* for 20 min. The supernatant was collected and mixed with Tyrode buffer containing 100 ng/ml prostaglandin E1 (PGE1, P5515, Sigma) in equal volumes and then centrifuged at 500 × *g* for 10 min at 24°C. Platelet precipitates were resuspended with Tyrode buffer supplemented with PGE1 (50 ng/ml) and ethylenediaminetetraacetic acid (EDTA, 1 mM), and the number of platelets was measured by an automatic blood cell analyzer (BC-5130, Mindray, Shenzhen).

### 2.5. Platelet Aggregation Assessment

The washed platelets were incubated with SAA1 (10 *μ*g/ml) for 5 min and then stimulated with thrombin (0.04 U/ml, SLBW3046, Sigma, USA), and platelet aggregation was measured by a lumi-aggregometer (CHRONO-LOG 700, Shanghai).

### 2.6. Adhesion of Platelets to Endothelial Cells

The washed platelets were incubated with calcein AM for 15 min, and HUVECs were treated with SAA1 (10 *μ*g/ml) or SAA1 (10 *μ*g/ml)+TAK-242 (1 *μ*g/ml) for 24 h, with the untreated HUVECs as the control. The preprocessed platelets were cocultured with those pretreated HUVECs for 1 h. After fixation with 4% paraformaldehyde for 40 min, the nuclei were stained by 4′,6-diamidino-2-phenylindole (DAPI, S2110, Solarbio, Beijing) for 10 min. The number of platelets adhering to endothelial cells was observed by inverted fluorescence microscopy.

### 2.7. Western Blot

HUVECs and THP-1 cells were lysed with RIPA (P0013B, Beyotime, China) containing 1% phenylmethylsulfonyl fluoride (PMSF, P0100, Solarbio, Beijing) and centrifuged at 4°C at 12,000 × *g* (ST16R, Thermo, Shanghai) for 20 min. Measurement of protein concentration was carried out using a bicinchoninic acid assay kit (P0010, Beyotime, China). After denaturation with a sample loading buffer (P0015, Beyotime, China), the proteins were separated by electrophoresis in sodium dodecyl sulfate-polyacrylamide gel electrophoresis (SDS-PAGE) gel (P0690, Beyotime, China) and transferred to polyvinylidene fluoride (PVDF) membranes (IPVH00010, Millipore). After inoculation with 5% skimmed milk powder (7BF0330, Yili, China) at room temperature for 4 h, PVDF membranes were incubated at 4°C with the following primary antibodies: MyD88 (1 : 2000, ab219413, Abcam, USA); P-IKK*α*/*β* (1 : 1500, ab194528, Abcam, USA); inhibitor of nuclear factor *κ*B kinase subunit *α*/*β* (IKK*α*/*β*, 1 : 1000, ab178870, Abcam, USA); P-IKB*α* (1 : 10000, ab133462, Abcam, USA); inhibitor of nuclear factor *κ*B subunit *α* (IKB*α*, 1 : 1000, ab76429, Abcam, USA); P-p65 (1 : 1000, ab76302, Abcam, USA); nuclear transcription factor-*κ*B p65 (p65, 1 : 2000, ab16502, Abcam, USA), or glyceraldehyde-3-phosphate dehydrogenase (GAPDH, 1 : 2000, D16H11, Cell Signalling Technology, USA) for 16 h and then incubated with horse radish peroxidase-labeled secondary antibodies (1 : 4000, ANR02-1, NeoBioscience) for 2 h. After washing with TBST, the protein band was then visualized by enhanced chemiluminescence (WBKLS0500, Millipore) using a chemiluminescence imager (CLINX5600, Clinx Science Instruments, Shanghai).

### 2.8. Real-Time PCR

HUVECs and THP-1 cells were lysed by TRIzol® reagent (A33250, Invitrogen, USA) to extract the total RNA. The cDNA was synthesized by MonScript™ RTIII All-in-One Mix with dsDNase (MR05101, Monad, China). The PCR reaction was performed with MonAmp™ SYBR® Green qPCR Mix (None/Low/High ROX) (MQ10101, Monad, China), with 40 cycles after predenaturation (30 sec at 95°C) followed by 10 sec at 95°C and 30 sec at 60°C. The aforementioned experiments were conducted according to the manufacturer's instructions. The expression level of GAPDH was used as an internal reference. The 2^-*ΔΔ*Cq^ method was used to quantify the relative gene expression [29]. Primer sequences (Beijing Qingke Biological Company) were as follows: GAPDH (human), sense (S) 5′-AATCCCATCACCATCTTCC-3′ and antisense (A) 5′-TTGAGGCTGTTGTCATACTTCT-3′; scavenger receptor A1 (SRA1, human), sense (S) 5′-ACTGATTGCCCTTTACCT-3′ and antisense (A) 5′-GTTGGCTTCCATGTCT AA-3′; cluster of differentiation 36 (CD36, human), sense (S) 5′-GTTTGGTTCCGTA CCCTG-3′ and antisense (A) 5′-CGATTATGGCAACTTTACTT-3′; LOX-1 (human), sense (S) 5′-GACCAGCCTGATGAGAAG-3′ and antisense (A) 5′-TCTGTCCCTCC AGTTTCT-3′; acyl-coenzyme A, cholesterol acyltransferase type 1 (ACAT1, human), sense (S) 5′-CAGGACGCTTATGCTATT-3′ and antisense (A) 5′-GCTGCTCCATCA TTCAGT-3′; ATP-binding cassette transporter A1 (ABCA1, human), sense (S) 5′-CTGCTAATTGCCAGACGG-3′ and antisense (A) 5′-GGTACTTGCCAAAGGGTG-3′; intercellular adhesion molecule 1 (ICAM1, human), sense (S) 5′-GCAAGAAGATAGCCAACCAA-3′ and antisense (A) 5′-TGCCAGTTCCACCCGTTC-3′; VCAM1 (human), sense (S) 5′-GGAATCTACAGCACCTTT-3′ and antisense (A) 5′-ACAGCCCATGACACTACA-3′; SELE (human), sense (S) 5′-GCACAGCCTTGTCCAACC-3′ and antisense (A) 5′-ACCTCACCAAACCCTTCG-3′; MCP-1 (human), sense (S) 5′-CTTCTGTGCCTGCTGCTC-3′ and antisense (A) 5′-TGCTGCTGGTGATTCTTCT-3′; tumor necrosis factor-*α* (TNF-*α*) (human), sense (S) 5′-TGTTGTAGCAAACCCTCAAGC-3′ and antisense (A) 5′-TGAAGAGGACCTGGGAGTAGAT-3′; interleukin-6 (IL-6) (human), sense (S) 5′-AGTAGTGAGGAACAAGCCAGAG-3′ and antisense (A) 5′-TACATTTGCCGAAGAGCC-3′; interleukin-1 beta (IL-1*β*, human), sense (S) 5′-ACAGTGGCAATGAGGATG-3′ and antisense (A) 5′-TGTAGTGGTGGTCGGA GA-3′; and chemokine (C-X-C motif) ligand 1 (CXCL1, human), sense (S) 5′-CCCCA AGAACATCCAAAGTG-3′ and antisense (A) 5′-GATGCAGGATTGAGGCAAG-3′.

### 2.9. Statistical Analysis

Data were analyzed using GraphPad Prism 8.3.0 (GraphPad Software, Inc.) and presented as the mean ± SEM of ≥3 independent experiments. The differences between the two groups were analyzed using an unpaired Student's *t*-test, and the differences among multiple groups were determined by one-way ANOVA with Tukey's post hoc test. Statistically significant differences were considered as *P* < 0.05.

## 3. Results

### 3.1. SAA1 Induces Inflammatory Activation of HUVECs

**Figure 1:**
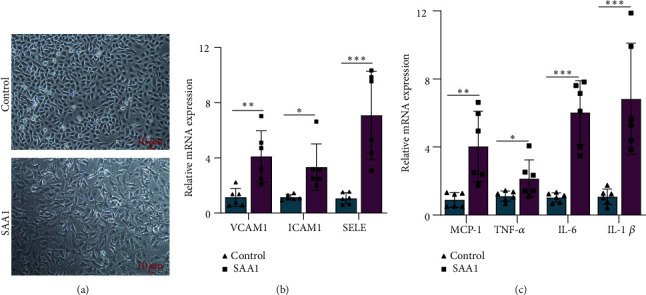
The effects of SAA1 on endothelial proinflammatory activation. (a) The morphology of HUVECs was observed under a microscope after treatment with or without SAA1. (b) Real-time PCR was used to detect the expression of adhesion molecules (VCAM1, ICAM1, and SELE) in HUVECs treated with or without SAA1. (c) Real-time PCR was used to detect the expression of proinflammatory molecules (MCP-1, TNF-*α*, IL-6, and IL-1*β*) in HUVECs treated with or without SAA1. Scale bar, 10 *μ*m. Data represent the mean ± SEM; *n* = 6 (^∗^*P* < 0.05, ^∗∗^*P* < 0.01, and ^∗∗∗^*P* < 0.001).

SAA1 is a stress protein with proinflammatory capacity [30, 31]. In this study, the effect of SAA1 on endothelial proinflammatory activation was evaluated through stimulating HUVECs with SAA1. As shown in Figure 1(a), HUVECs contracted and their processes became elongated due to stimulation with SAA1. SAA1 stimulation elevated the expression of VCAM1, ICAM1, SELE, MCP-1, TNF-*α*, IL-6, and IL-1*β* in HUVECs (Figures 1(b) and 1(c) and Supplementary Table 1). These results suggest that SAA1 plays a positive role in promoting the inflammatory activation of endothelial cells.

### 3.2. SAA1 Promotes the Formation of Foam Cells

**Figure 2:**
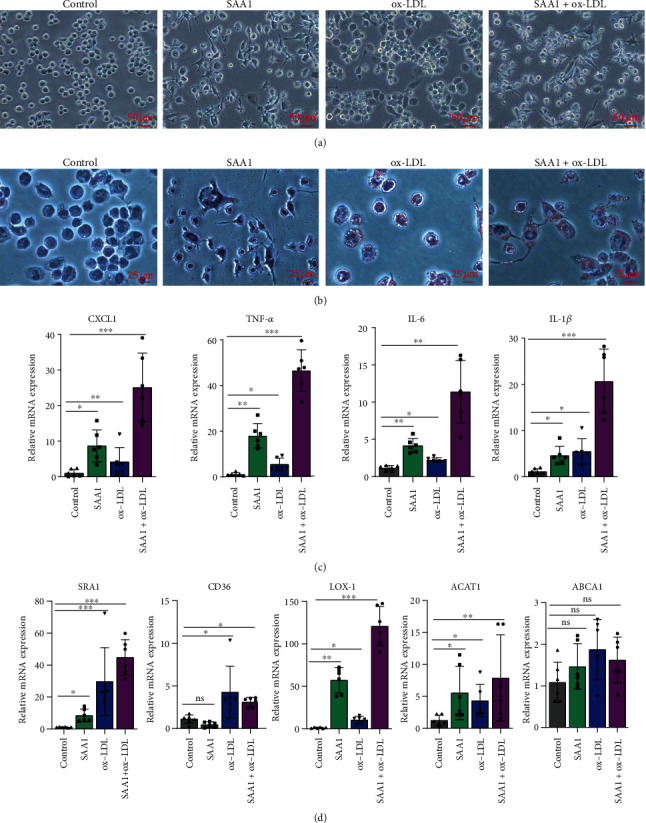
Effects of SAA1 on activation of macrophages and foam-cell formation. The macrophages derived from THP-1 cells induced by PMA were treated with SAA1, ox-LDL, or SAA1+ox-LDL, with the untreated macrophages as the control. (a) The morphology of macrophages was observed under microscopy. (b) Lipid deposition in cells was assessed by Oil Red O staining. (c) The expression of CXCL1, TNF-*α*, IL-6, and IL-1*β* was detected by real-time PCR. (d) The expression of SRA1, CD36, LOX-1, ACAT1, and ABCA1 was detected by real-time PCR. Scale bars, 25 *μ*m and 50 *μ*m. Data represent the mean ± SEM; *n* = 6 (ns: not significant; ^∗^*P* < 0.05, ^∗∗^*P* < 0.01, and ^∗∗∗^*P* < 0.001).

The formation of foam cells is one of the major markers of atherosclerosis [32]. In previous studies [33], ox-LDL was often used to induce the differentiation of monocyte- derived macrophages into foam cells. Here, to investigate the effect of SAA1 on activation of macrophages and foam-cell formation, macrophages derived from THP-1 cells induced by PMA were treated with SAA1, ox-LDL, or SAA1+ox-LDL. As shown in Figure 2(a), THP-1-derived macrophages stimulated by SAA1 formed more cell processes than the cells stimulated by ox-LDL, while macrophages stimulated by SAA1+ox-LDL formed more cell processes than those stimulated by SAA1 or ox-LDL, respectively. More lipid deposition and higher mRNA levels of inflammatory molecules (CXCL1, TNF-*α*, IL-6, and IL-1*β*) were observed in macrophages stimulated with SAA1+ox-LDL than those stimulated with SAA1 or ox-LDL, respectively (Figures 2(b) and 2(c) and Supplementary Table 2). In addition, compared with ox-LDL-stimulated macrophages, the higher expression levels of the foam-cell formation-related molecule LOX-1 were induced in macrophages stimulated with SAA1+ox-LDL, while other foam-cell formation-related molecules, SRA1, CD36, ACAT1, and ABCA1 showed no significant difference (Figure 2(d) and Supplementary Table 2). These results imply that SAA1 could promote the formation of foam cells, and it is possible to achieve this function by inducing the expression of LOX-1.

### 3.3. SAA1 Accelerates Monocyte Adhesion to Endothelial Cells

**Figure 3:**
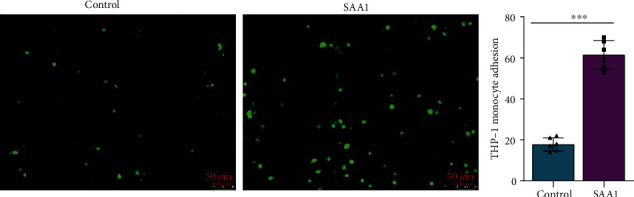
The effects of SAA1 on monocyte adhesion to endothelial cells. (a) THP-1 cells preincubated with calcein AM for 15 min were cocultured with HUVECs pretreated with or without SAA1 for 24 h. Six hours later, the number of THP-1 cells adhering to endothelial cells was observed by fluorescence microscopy. Scale bar, 50 *μ*m. Data represent the mean ± SEM; *n* = 6 (^∗∗∗^*P* < 0.001).

Adhesion of monocytes to endothelial cells is one of the important pathological processes in atherosclerosis [34, 35]. To further investigate whether SAA1 promotes monocyte adhesion to endothelial cells, THP-1 cells preincubated with calcein AM were cocultured with HUVECs pretreated with or without SAA1. It was shown that more THP-1 cells were adhered to SAA1 pretreated HUVECs (Figure 3(a) and Supplementary Table 3). These results demonstrate that SAA1 promotes monocyte adhesion to endothelial cells.

### 3.4. SAA1 Promotes Platelet Aggregation and Platelet Adhesion to Endothelial Cells

**Figure 4:**
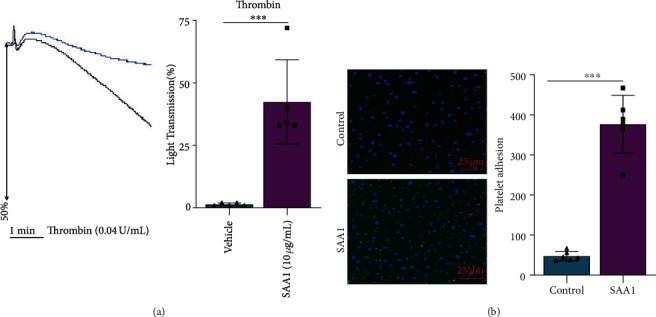
Effects of SAA1 on platelet aggregation and platelet adhesion to endothelial cells. (a) Platelets preincubated with or without SAA1 (10 *μ*g/ml) were stimulated with thrombin (0.04 U/ml), and platelet aggregation was detected by an aggregator. (b) Platelets preincubated with calcein AM for 15 min were cocultured with HUVECs pretreated with or without SAA1 for 24 h. One hour later, the number of platelets adhering to endothelial cells was observed by fluorescence microscopy. Data represent the mean ± SEM; *n* = 6 (^∗∗∗^*P* < 0.001).

Platelet aggregation and adhesion to endothelial cells contribute to atherosclerosis [36]. In this study, platelets were preincubated with SAA1 and then stimulated with thrombin, and SAA1 significantly promoted platelet aggregation (Figure 4(a) and Supplementary Table 4). In addition, platelet preincubated with calcein AM was cocultured with HUVECs pretreated with or without SAA1. One hour later, it was observed that more platelets were adhered to SAA1 pretreated HUVECs (Figure 4(b) and Supplementary Table 5). These results demonstrate that SAA1 promotes platelet aggregation and platelet adhesion to endothelial cells.

### 3.5. TLR4 Inhibitors Reduce SAA1-Induced Endothelial Inflammatory Activation

**Figure 5:**
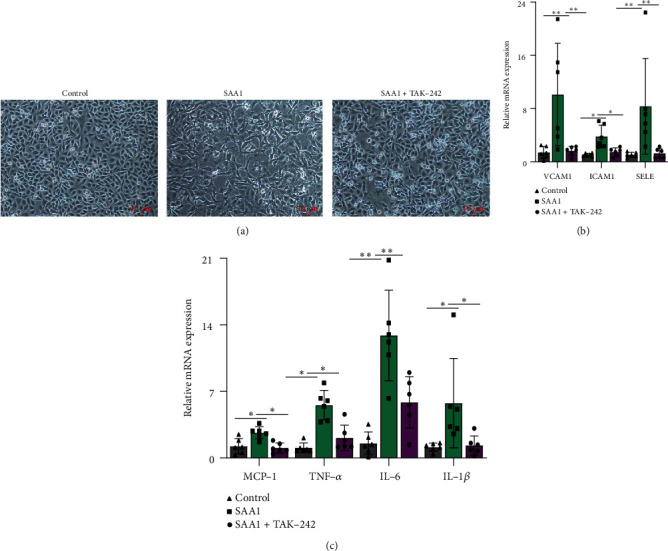
The role of TLR4 in SAA1-induced endothelial inflammatory phenotype. HUVECs were stimulated with SAA1 or SAA1+TAK-242, respectively. (a) Morphological changes in cells were observed by microscopy. (b) The expression of adhesion molecules (VCAM1, ICAM1, and SELE) was detected by real-time PCR. (c) Real-time PCR was used to detect the expression of proinflammatory molecules (MCP-1, TNF-*α*, IL-6, and IL-1*β*). Scale bar, 10 *μ*m. Data represent the mean ± SEM; *n* = 6 (^∗^*P* < 0.05 and ^∗∗^*P* < 0.01).

TLR4 signaling activation is one of the important factors leading to the inflammatory activation of endothelial cells, and inhibition of TLR4 signaling will weaken this inflammatory activation [27, 37, 38]. In order to explore the role of TLR4 in the inflammatory phenotype of endothelial cells induced by SAA1, HUVECs were treated with SAA1 or SAA1+TAK-242. It was observed that, compared with the HUVECs treated with SAA1, cellular contraction and elongation of cell processes were reduced in HUVECs treated with SAA1+TAK-242 (Figure 5(a)), and the expression of adhesion molecules (VCAM1, ICAM1, and SELE) and proinflammatory molecules (MCP-1, TNF-*α*, IL-6, and IL-1*β*) was also significantly decreased (Figures 5(b) and 5(c) and Supplementary Table 6). It can be seen from the above results that TLR4 plays an important role in the SAA1-induced endothelial inflammatory phenotype.

### 3.6. TLR4 Plays an Important Role in SAA1-Induced Macrophage Activation and Foam-Cell Formation

**Figure 6:**
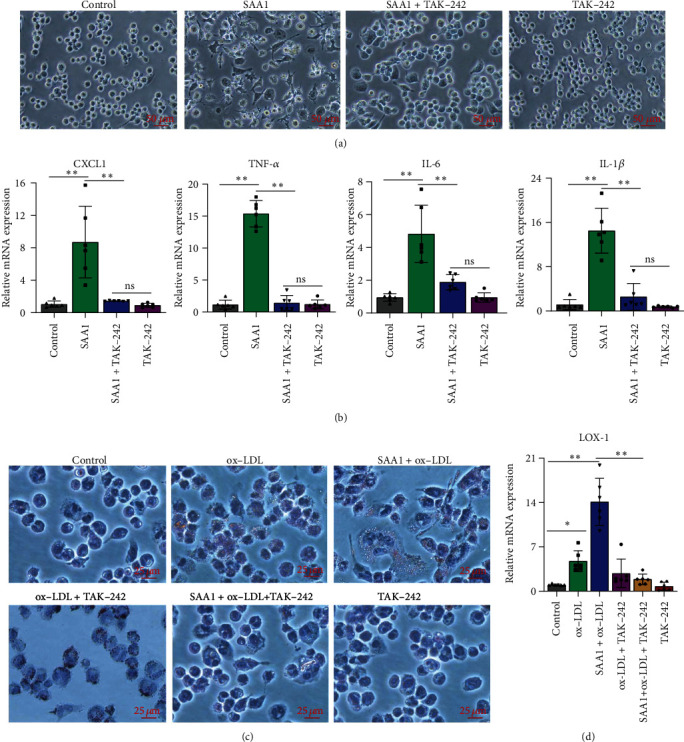
The role of TLR4 in SAA1-induced macrophage activation and foam-cell formation. The macrophages derived from THP-1 cells induced by PMA were treated with SAA1, TAK-242, or SAA1+TAK-242, with the untreated macrophages as the control. (a) Cell morphology was observed under a microscope. (b) The expression of CXCL1, TNF-*α*, IL-6, and IL-1*β* was detected by real-time PCR. (c) The macrophages derived from THP-1 cells induced by PMA were treated with ox-LDL, SAA1, SAA1+ox-LDL, ox-LDL+TAK-242, SAA1+ox-LDL+TAK-242, or TAK-242, with the untreated macrophages as the control. Intracellular lipid deposition was observed by oil red O staining. (d) The expression of LOX-1 was detected by real-time PCR. Scale bar, 25 *μ*m. Data represent the mean ± SEM; *n* = 6 (ns: not significant; ^∗^*P* < 0.05 and ^∗∗^*P* < 0.01).

Activation of TLR4 signaling not only plays a key role in inducing polarization of macrophages toward inflammatory phenotype but also promotes the formation of foam cells [28]. Here, we further explored whether SAA1-induced macrophage activation and foam-cell formation depended on TLR4 signaling. As shown in Figures 6(a) and 6(b)and Supplementary Table 7, compared with SAA1-stimulated macrophages, macrophages stimulated with SAA1+TAK-242 formed fewer cell processes, and they expressed lower proinflammatory molecules (CXCL1, TNF-*α*, IL-6, and IL-1*β*). In addition, TLR4 inhibitor (TAK-242) also significantly inhibited lipid deposition and LOX-1 expression in SAA1+ox-LDL-induced macrophages (Figures 6(c) and 6(d) and Supplementary Table 8). These results further suggest that TLR4 signaling is vital in SAA1-induced macrophage activation and foam-cell formation.

### 3.7. TLR4 Plays an Important Role in SAA1-Induced Monocyte and Platelet Adhesion to Endothelial Cells

**Figure 7:**
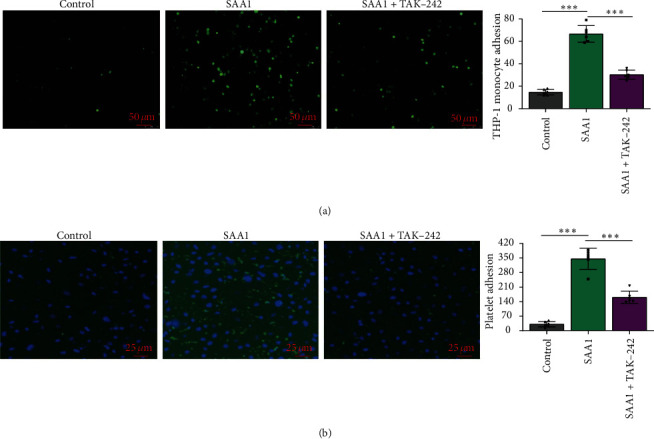
The role of TLR4 in SAA1-induced monocyte and platelet adhesion to endothelial cells. HUVECs were treated with SAA1 or SAA1+TAK-242 for 24 h, with the untreated HUVECs as the control. These HUVECs were used for coculture with THP-1 cells/platelets preincubated with calcein AM for 15 min. (a) Six hours later, the number of THP-1 cells adhering to endothelial cells was observed by fluorescence microscopy. (b) After coculture with platelets for 1 h, the nuclei of HUVECs were stained by DAPI. Scale bar, 25 *μ*m and 50 *μ*m. Data represent the mean ± SEM; *n* = 6 (^∗∗∗^*P* < 0.001).

Based on the above studies, we further investigated the role of TLR4 in SAA1-induced adhesion of monocytes and platelets to endothelial cells. As shown in Figures 7(a) and 7(b) and Supplementary Tables 9-10, compared with endothelial cells prestimulated by SAA1, the number of monocytes and platelets adhering to endothelial cells prestimulated by SAA1+TAK-242 decreased significantly. These results indicate that SAA1-induced adhesion of monocytes and platelets to endothelial cells may depend on TLR4.

### 3.8. TLR4 Inhibitor Restrains the Activation of NF-*κ*B Signaling in SAA1-Induced HUVECs and Macrophages

**Figure 8:**
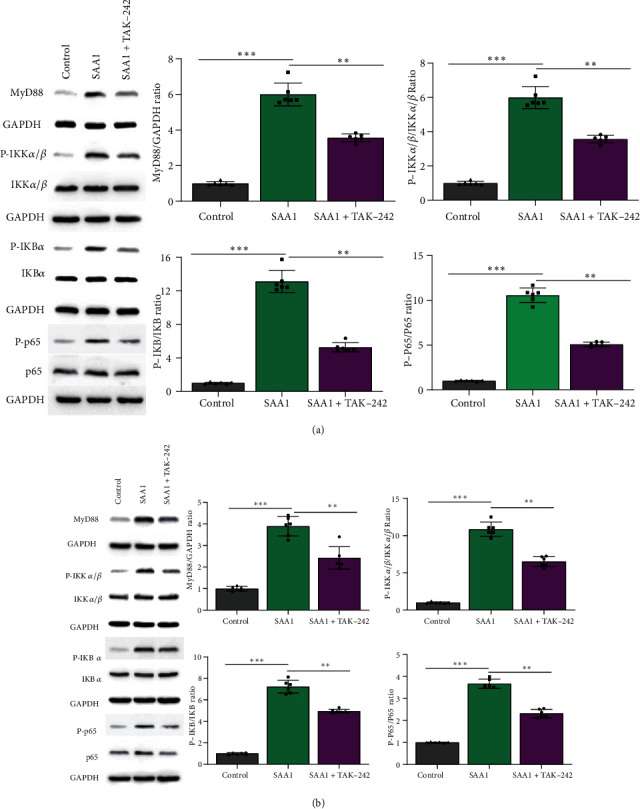
TLR4 inhibitor restrains the activation of NF-*κ*B signaling in SAA1-induced HUVECs and macrophages. (a) The expression of MyD88, P-IKK*α*/*β*, IKK*α*/*β*, P-IKB*α*, IKB*α*, P-p65, and p65 was detected by Western blot after HUVECs were treated with SAA1 or SAA1+TAK-242, with the untreated HUVECs as the control. (b) The macrophages derived from THP-1 induced by PMA were treated with SAA1 or SAA1+TAK-242, with the untreated macrophages as the control, and then the expression of MyD88, P-IKK*α*/*β*, IKK*α*/*β*, P-IKB*α*, IKB*α*, P-p65, and p65 was detected by Western blot. Data represent the mean ± SEM; *n* = 6 (^∗∗^*P* < 0.01 and ^∗∗∗^*P* < 0.001).

NF-*κ*B signaling is a major downstream signal for TLR4 [39, 40]. Therefore, we further explored whether SAA1 could activate NF-*κ*B signaling through TLR4 and induce the expression of NF-*κ*B signaling-related molecules. As shown in Figures 8(a) and 8(b) and Supplementary Tables 11-12, SAA1 significantly promoted the expression of NF-*κ*B signaling molecules MyD88, P-IKK*α*/*β*, P-IKB*α*, and P-p65, while TLR4 inhibitor restrained the expression of NF-*κ*B signaling molecules induced by SAA1. These results suggest that SAA1-induced NF-*κ*B signaling activation depends on TLR4 in HUVECs and macrophages.

## 4. Discussion

As the main pathological process of cardiovascular diseases, including coronary heart disease and stroke, the mechanism of atherosclerosis has been continuously explored by a large number of researchers [41]. Although the prevailing view supports that lipid-driven disease is caused by local accumulation of LDL and residual lipoprotein particles in the middle and large arteries, a growing number of researchers have also demonstrated that lipid-induced inflammatory signaling plays a role in atherosclerosis [42]. These inflammatory signals contain not only inflammatory factors secreted by immune cells but also other proinflammatory proteins induced by abnormal lipid metabolism [1]. For example, it has been confirmed that lipocalin-2, a proinflammatory protein, promotes atherosclerosis by enhancing M1-type macrophage polarization, foam-cell formation, and inflammatory activation of endothelial cells [43]. Motivated by these new findings and theories, we hypothesized and explored the function and possible regulatory mechanisms of SAA1, an inflammatory stress protein, on platelet aggregation, endothelial cell inflammatory activation, foam-cell formation, and monocyte/platelet adhesion to endothelial cells.

The pathogenesis of atherosclerosis is, in part, related to dyslipidemia and hypercholesterolemia, which can be triggered by genetic factors, diet and lifestyle choices, and metabolic disorders such as obesity and type 2 diabetes (T2D) [44]. SAA1 expression is increased in high-fat diet-induced obesity and T2D and is closely associated with obesity-induced metabolic syndrome-related complications [20, 45]. In fact, various studies have not only confirmed the important role of SAA1 in diseases related to the formation of atherosclerosis but also showed the potential role of SAA1 in atherosclerosis to varying degrees [21, 46]. For example, Thompson et al. found in their study that SAA1 induced vascular smooth muscle cells to secrete matrix proteoglycans [21], and Lee et al. showed that SAA1 promoted the formation of foam cells by inducing the expression of LOX-1 [47]. Moreover, Dong et al. found that SAA could promote the expression of VCAM1 in endothelial cells, and SAA directly accelerated the progression of atherosclerosis in ApoE^−/−^ mice [48]. Therefore, on the basis of these studies, a series of in vitro experiments were conducted to further systematically confirm the promotive role of SAA1 in regulating platelet aggregation, inflammatory activation of endothelial cells, and foam-cell formation. The role of SAA1 in enhancing monocyte/platelet adhesion to endothelial cells was also proved, and these findings extended the regulatory role of SAA1 in promoting atherosclerosis.

Numerous studies have shown that SAA1 regulates the biological functions of various cells principally by binding with its potential receptors or other effector molecules [13]. In particular, potential receptors of SAA1 have been identified in immune cells, including P2RX7, CD36, recombinant Scavenger Receptor Class B Member 1 (SCARB1), valosin-containing protein (VCP), and TLR2/4 [14, 23, 49–52]. For example, SAA1 activates the nucleotide-binding domain leucine-rich repeat (NLR) family pyrin domain containing 3 (NLRP3) inflammasome via P2RX7, leading to inflammatory activation and IL-1*β* secretion of macrophages [53]. SAA1 also activated the CD36 receptor of rat macrophages and promoted the expression of IL-6 and TNF-*α* [54]. In mouse macrophages, SAA1 promotes the expression of proinflammatory cytokines (interleukin-23*α* (IL-23*α*) and TNF-*α*) through TLR2 and increases the level of nitric oxide through TLR4 [55–57]. Furthermore, SAA1 induces IL-6 expression through TLR2/4-related signaling and activates NF-*κ*B signaling in fibroblasts [58, 59]. These results indicate that the proinflammatory function of SAA1 depends on its related receptors and downstream signaling. The present study not only confirmed that the role of SAA1 in promoting endothelial cells activation, foam-cell formation, and monocyte/platelet adhesion to endothelial cells might depend on TLR4 but also proved that SAA1 activated NF-*κ*B signaling through TLR4 in endothelial cells and monocytes. It has been demonstrated that the NF-*κ*B signaling pathway is vital for endothelial cell activation and foam-cell formation. Our results are also consistent with previous reports that SAA1 activates NF-*κ*B signaling in other cells [18, 58, 60].

Here, based on in vitro evidence that SAA1 promotes atherosclerosis, we further confirm that such function of SAA1 may depend on activation of the TLR4-related NF-*κ*B signaling pathway. However, our study also raises some scientific questions: first, what are the mechanisms that lead to increased SAA1 expression in atherosclerotic diseases (metabolic syndrome, obesity and diabetes), inflammatory, or other factors? Second, in addition to platelets, endothelial cells, and monocytes, other cells (dendritic cells, T cells, vascular smooth muscle cells, mast cells, and neutrophils) are indirectly involved in the development of atherosclerosis [1], so it is not clear whether SAA1 may also aggravate atherosclerosis by affecting the biological functions of these cells. Third, there are many potential receptors for SAA1, and our study clarified only the potential role of TLR4 signaling in SAA1-induced atherosclerosis; whether other receptors also play corresponding functions needs to be further explored. Fourth, there may be clinical value in seeking new pharmacologic inhibitors against the functional activity of SAA1 to block the formation of atherosclerosis. These scientific issues above will be the focus for further efforts in future research.

## 5. Conclusion

SAA1 could promote atherosclerosis through enhancing endothelial cell activation, foam-cell formation, platelet aggregation, and monocyte/platelet adhesion to endothelial cells. Such biological functions of SAA1 might depend on the activation of the TLR4-related NF-*κ*B signaling pathway.

## Data Availability

The data used to support the findings of this study are available from the corresponding author upon request.
