# Correction to “Regulation of Atherosclerosis by Toll‐Like Receptor 4 Induced by Serum Amyloid 1: A Systematic In Vitro Study”

**DOI:** 10.1155/bmri/9803273

**Published:** 2026-06-12

J. Chen, G. Liu, Y. Hong, et al., “Regulation of Atherosclerosis by Toll‐Like Receptor 4 Induced by Serum Amyloid 1: A Systematic In Vitro Study,” *BioMed Research International*, 2022, 4887593, 10.1155/2022/4887593

In the article, the authors have identified errors in the blots shown in Figure 8b. Specifically:

• The P‐p65 bands in Figures 8a and 8b are identical
• The bottom two sets of GAPDH bands in Figure 8b are identical
• The p65 bands in 8b are incorrect

After assessment by the editorial board of the concerns and the author’s original images, it has been confirmed that the results and conclusions are unaffected by the errors. The revised Figure 8 is shown below, which corrects the GAPDH, P‐p65 and p65 bands in Figure 8b:

**Figure 8:**
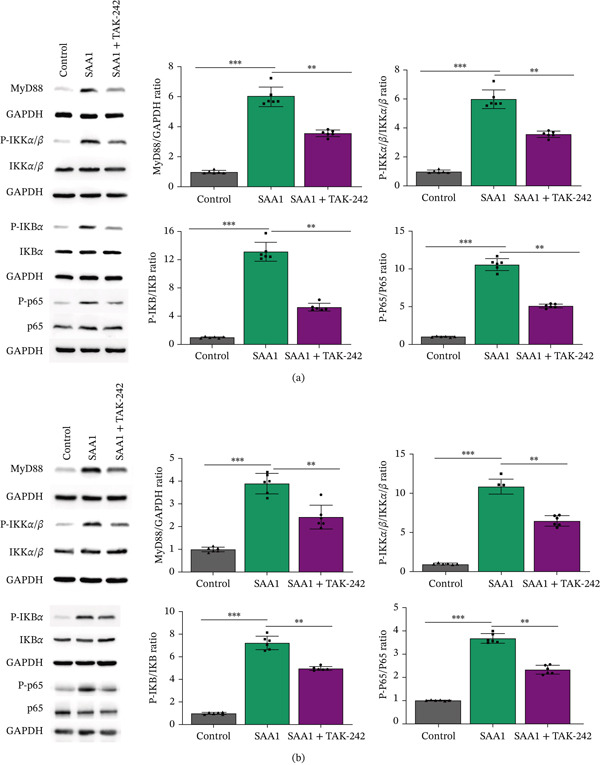
TLR4 inhibitor restrains the activation of NF‐*κ*B signaling in SAA1‐induced HUVECs and macrophages. (a) The expression of MyD88, P‐IKK*α*/*β*, IKK*α*/*β*, P‐IKB*α*, IKB*α*, P‐p65, and p65 was detected by Western blot after HUVECs were treated with SAA1 or SAA1 + TAK‐242, with the untreated HUVECs as the control. (b) The macrophages derived from THP‐1 induced by PMA were treated with SAA1 or SAA1 + TAK‐242, with the untreated macrophages as the control, and then the expression of MyD88, P‐IKK*α*/*β*, IKK*α*/*β*, P‐IKB*α*, IKB*α*, P‐p65, and p65 was detected by Western blot. Data represent the mean ± SEM; *n* = 6 (∗∗*p* < 0.01 and ∗∗∗*p* < 0.001).

We apologize for these errors.

