# Supplementary material for: Regulation of Atherosclerosis by Toll-Like Receptor 4 Induced by Serum Amyloid 1: A Systematic In Vitro Study
Source: Biomed Res Int. 2022 Sep 15;2022:4887593. doi: 10.1155/2022/4887593 (PMC9499805; doi:10.1155/2022/4887593)
Supplement: Supplementary Materials — Supplementary data to this article can be found in supplementary information. [file 4887593.f1.docx]

**Supplementary Information**

**Supplementary table 1 (corresponding to figure 1b-c).** Expression analysis of adhesion molecules and pro-inflammatory molecules in HUVECs treated with or without SAA1.

|  | Control | SAA1 | *F* | *P* |
| --- | --- | --- | --- | --- |
| VCAM1 | 1.14±0.65 | 4.10±1.86^**^ | 7.134 | <0.01 |
| ICAM1 | 1.14±0.19 | 3.33±1.68^*^ | 3.856 | <0.05 |
| SELE | 1.06±0.41 | 7.08±3.20^***^ | 61.883 | <0.001 |
| MCP-1 | 0.89±0.43 | 4.04±2.07^**^ | 34.785 | <0.01 |
| TNF-α | 1.10±0.32 | 2.48±0.87^*^ | 1.277 | <0.05 |
| IL-6 | 1.03±0.27 | 6.03±1.88^***^ | 13.803 | <0.001 |
| IL-1β | 1.09±0.45 | 6.83±3.27^***^ | 17.312 | <0.001 |

Note: real-time PCR was used to detect the expression of adhesion molecules (VCAM1, ICAM1 and SELE) and pro-inflammatory molecules (MCP-1, TNF-α, IL-6 and IL-1β) in HUVECs treated with or without SAA1. Data were analyzed using GraphPad Prism 8.3.0, the differences between the two groups were analyzed using an unpaired Student’s t-test. ^*^means compared with the control group, n=6 (^*^*P*<0.05; ^**^*P*<0.01; ^***^*P*<0.001).

**Supplementary table 2 (corresponding to figure 2c-d).** Expression analysis of pro-inflammatory molecules and foam-cell formation-related molecules in macrophages derived from THP-1 cells following treatment with SAA1, ox-LDL or SAA1+ox-LDL.

|  | Control | SAA1 | ox-LDL | SAA1+ox-LDL | *F* | *P* |  |
| --- | --- | --- | --- | --- | --- | --- | --- |
| CXCL 1 | 1.04±1.03 | 8.71±4.42^**^ | 4.21±4.00^*^ | 25.10±9.63^***^ | 21.215 | <0.05 |  |
| TNF-α | 1.13±0.68 | 17.96±5.35^**^ | 5.51±2.68^*^ | 46.53±9.15^***^ | 83.643 | <0.05 |  |
| IL-6 | 1.13±0.33 | 4.15±0.95^**^ | 2.23±0.31^*^ | 11.40±4.20^***^ | 27.423 | <0.05 |  |
| IL-1β | 1.13±0.62 | 4.62±1.98^**^ | 5.50±2.74^*^ | 20.66±6.95^***^ | 29.937 | <0.05 |  |
| SRA 1 | 1.07±0.26 | 8.49±3.93^*^ | 29.76±21.25^*^ | 44.92±11.00^***^ | 16.354 | <0.05 |  |
| CD36 | 1.12±0.47 | 0.47±0.32 | 4.27±3.04^*^ | 3.11±0.56^*^ | 7.502 | <0.05 |  |
| LOX-1 | 1.17±0.65 | 57.22±14.80^*^ | 10.52±4.11^*^ | 120.84±23.20^***, #^ | 92.200 | <0.05 |  |
| ACAT 1 | 1.26±0.80 | 5.55±4.18^*^ | 4.32±2.55^*^ | 8.45±6.23^*^ | 3.358 | <0.05 |  |
| ABCA 1 | 1.08±0.48 | 1.47±0.55 | 1.87±0.720 | 1.62±0.55 | 1.938 | >0.05 |  |

Note: the macrophages derived from THP-1 cells induced by PMA were treated with SAA1, ox-LDL or SAA1+ox-LDL, with the untreated macrophages as the control. The expression of CXCL1, TNF-α, IL-6, IL-1β, SRA1, CD36, LOX-1 and ACAT1 was detected by real-time PCR. Data were analyzed using GraphPad Prism 8.3.0, the differences among multiple groups were determined by one-way ANOVA with Tukey’s post hoc test. ^*^mean compared with the control group; ^#^means ox-LDL compared with SAA1+ox-LDL, n=6 (^*^*P<*0.05; ^**^*P*<0.01; ^***^*P*<0.001; ^#^*P<*0.05).

**Supplementary table 3 (corresponding to figure 3a).** Statistical analysis of the number of THP-1 cells adhering to endothelial cells pretreated with or without SAA1.

|  | Control | SAA1 | *F* | *P* |
| --- | --- | --- | --- | --- |
| THP-1 adhesion | 17.8±3.20 | 61.50±6.95^***^ | 6.579 | <0.001 |

Note: THP-1 cells pre-incubated with calcein AM for 15 min were co-cultured with HUVECs pretreated with or without SAA1 for 24 h. Six hours later, the number of THP-1 cells adhering to endothelial cells was observed by fluorescence microscopy. Data were analyzed using GraphPad Prism 8.3.0, the differences between the two groups were analyzed using an unpaired Student’s t-test. ^*^means compared with the control group, n=6 (^***^*P*<0.001).

**Supplementary table 4 (corresponding to figure 4a).** Statistical analysis of platelet aggregation.

|  | Control | SAA1 | *F* | *P* |
| --- | --- | --- | --- | --- |
| Light transmission | 1.40±0.55 | 42.4±16.80^***^ | 6.026 | <0.001 |

Note: platelets pre-incubated with or without SAA1 (10 μg/mL) were stimulated with thrombin (0.04 U/ mL), and platelet aggregation was detected by an aggregator. Data were analyzed using GraphPad Prism 8.3.0, the differences between the two groups were analyzed using an unpaired Student’s t-test. ^*^means compared with the control group, n=5 (^***^*P*<0.001).

**Supplementary table 5 (corresponding to figure 4b).** Statistical analysis of the number of platelets adhering to endothelial cells pretreated with or without SAA1.

|  | Control | SAA1 | *F* | *P* |
| --- | --- | --- | --- | --- |
| platelet adhesion | 47.50±11.84 | 367.50±72.10^***^ | 3.089 | <0.001 |

Note: platelets pre-incubated with calcein AM for 15 min were co-cultured with HUVECs pretreated with or without SAA1 for 24 h. One hour later, the number of platelets adhering to endothelial cells was observed by fluorescence microscopy. Data were analyzed using GraphPad Prism 8.3.0, the differences between the two groups were analyzed using an unpaired Student’s t-test. ^*^means compared with the control group, n=6 (^***^*P*<0.001).

**Supplementary table 6 (corresponding to figure 5b-c).** Expression analysis of adhesion molecules and pro-inflammatory molecules in HUVECs stimulated with SAA1 or SAA1+TAK-242.

|  | Control | SAA1 | SAA1+TAK-242 | *F* | *P* |
| --- | --- | --- | --- | --- | --- |
| VCAM 1 | 1.39±0.90 | 10.08±7.70^*^8 | 1.63±0.68^##^ | 7.291 | <0.05 |
| ICAM 1 | 1.05±0.23 | 3.80±1.70^*^ | 1.47±0.62^#^ | 11.873 | <0.05 |
| SELE | 1.06±0.37 | 8.33±7.20^**^ | 1.25±0.74^##^ | 5.897 | <0.05 |
| MCP-1 | 1.24±0.80 | 2.66±0.64^*^ | 1.06±0.52^#^ | 10.380 | <0.05 |
| TNF-α | 1.07±0.51 | 5.56±1.55^*^ | 2.11±1.35^#^ | 22.086 | <0.05 |
| IL-6 | 1.54±1.20 | 12.88±4.76^**^ | 5.854±2.69^*, ##^ | 18.862 | <0.05 |
| IL-1β | 1.13±0.43 | 5.77±4.70^*^ | 1.31±1.00^#^ | 5.331 | <0.05 |

Note: real-time PCR was used to detect the expression of adhesion molecules (VCAM1, ICAM1 and SELE) and pro-inflammatory molecules (MCP-1, TNF-α, IL-6 and IL-1β) in HUVECs stimulated with SAA1 or SAA1+TAK-242. Data were analyzed using GraphPad Prism 8.3.0, the differences among multiple groups were determined by one-way ANOVA with Tukey’s post hoc test. ^*^means compared with the control group; ^#^means SAA1 compared with SAA1+TAK-242, n=6 (^*^*P<*0.05; ^**^*P*<0.01; ^#^*P<*0.05; ^##^*P<*0.01).

**Supplementary table 7 (corresponding to figure 6b)**. Expression analysis of pro-inflammatory molecules in macrophages derived from THP-1 cells following treatment with SAA1, TAK-242 or SAA1+TAK-242.

|  | Control | SAA1 | SAA1+TAK-242 | TAK-242 | *F* | *P* |
| --- | --- | --- | --- | --- | --- | --- |
| CXCL1 | 1.05±0.40 | 8.71±4.42^**^ | 1.45±0.42^##^ | 0.92±0.24 | 17.410 | <0.01 |
| TNF-α | 1.16±0.69 | 15.40±2.06^**^ | 1.43±1.17^##^ | 1.22±0.69 | 182.373 | <0.01 |
| IL-6 | 0.94±0.23 | 4.82±1.75^**^ | 1.86±0.46^##^ | 0.94±0.29 | 23.692 | <0.01 |
| IL-1β | 1.18±0.91 | 14.51±4.05^**^ | 2.57±2.42^##^ | 0.77±0.14 | 44.558 | <0.01 |

Note: the macrophages derived from THP-1 cells induced by PMA were treated with SAA1, TAK-242 or SAA1+TAK-242, with the untreated macrophages as the control. The expression of CXCL1, TNF-α, IL-6 and IL-1β was detected by real-time PCR. Data were analyzed using GraphPad Prism 8.3.0, the differences among multiple groups were determined by one-way ANOVA with Tukey’s post hoc test. ^*^means compared with the control group; ^#^means SAA1 compared with SAA1+TAK-242, n=6 (^**^*P*<0.01; ^##^*P<*0.01).

**Supplementary table 8 (corresponding to figure 6d).** Expression analysis of LOX-1 in macrophages derived from THP-1 cells following treatment with ox-LDL, SAA1+ox-LDL, ox-LDL+TAK-242, SAA1+ox-LDL+TAK-242 or TAK-242.

|  | LOX-1 |
| --- | --- |
| Control | 1.01±0.11 |
| ox-LDL | 4.74±1.63^*^ |
| SAA1+ox-LDL | 14.12±3.72^**^ |
| ox-LDL+TAK-242 | 2.82±2.26 |
| SAA1+ox-LDL+TAK-242 | 1.88±0.82^##^ |
| TAK-242 | 0.77±0.65 |
| *F* | 40.587 |
| *P* | P<0.05 |

Note: the macrophages derived from THP-1 cells induced by PMA were treated with ox-LDL, SAA1, SAA1+ox-LDL, ox-LDL+TAK-242, SAA1+ox-LDL+TAK-242 or TAK-242, with the untreated macrophages as the control. The expression of LOX-1 was detected by real-time PCR. Data were analyzed using GraphPad Prism 8.3.0, the differences among multiple groups were determined by one-way ANOVA with Tukey’s post hoc test. ^*^means compared with the control group; ^#^means SAA1+ox-LDL+TAK-242 compared with SAA1+TAK-242, n=6 (^*^*P*<0.05; ^**^*P*<0.01; ^##^*P<*0.01).

**Supplementary table 9 (corresponding to figure 7a).** Statistical analysis of the number of THP-1 cells adhering to endothelial cells stimulated with SAA1 or SAA1+ TAK-242.

|  | Control | SAA1 | SAA1+TAK-242 | *F* | *P* |
| --- | --- | --- | --- | --- | --- |
| THP-1 adhesion | 14.67±2.50 | 66.67±7.42^***^ | 30.33±4.03^###^ | 165.060 | <0.001 |

Note: HUVECs were treated with SAA1 or SAA1+TAK-242 for 24 h, with the untreated HUVECs as the control. These HUVECs were used for co-culture with THP-1 cells pre-incubated with calcein AM for 15 min. Six hours later, the number of THP-1 cells adhering to endothelial cells was observed by fluorescence microscopy. Data were analyzed using GraphPad Prism 8.3.0, the differences among multiple groups were determined by one-way ANOVA with Tukey’s post hoc test. ^∗^means compared with the control group; ^#^means SAA1 compared with SAA1+TAK-242, n=6 (^***^*P*<0.001; ^###^*P<*0.001).

**Supplementary table 10 (corresponding to figure 7b).** Statistical analysis of the number of platelets adhering to endothelial cells stimulated with SAA1 or SAA1+ TAK-242.

|  | Control | SAA1 | SAA1+TAK-242 | *F* | *P* |
| --- | --- | --- | --- | --- | --- |
| Platelet adhesion | 31.67±13.11 | 364.33±50.29^***^ | 161.17±29.31^###^ | 126.440 | <0.001 |

Note: HUVECs were treated with SAA1 or SAA1+TAK-242 for 24 h, with the untreated HUVECs as the control. These HUVECs were used for co-culture with platelets pre-incubated with calcein AM for 15 min. One hour later, the number of platelets adhering to endothelial cells was observed by fluorescence microscopy. Data were analyzed using GraphPad Prism 8.3.0, the differences among multiple groups were determined by one-way ANOVA with Tukey’s post hoc test. ^*^means compared with the control group; ^#^means SAA1 compared with SAA1+TAK-242, n=6 (^***^*P*<0.001; ^###^*P<*0.001).

**Supplementary table 11 (corresponding to figure 8a).** Expression analysis of MyD88, P-IKKα/β, IKKα/β, P-IKBα, IKBα, P-p65 and p65 in HUVECs treated with SAA1 or SAA1+TAK-242.

|  | Control | SAA1 | SAA1+TAK-242 | *F* | *P* |
| --- | --- | --- | --- | --- | --- |
| MyD88 | 1.00±0.10 | 6.00±0.640^***^ | 3.56±0.22^##^ | 240.861 | <0.05 |
| P-IKKα/β/IKKα/β | 1.00±0.11 | 6.03±0.47^***^ | 3.81±0.42^##^ | 277.481 | <0.05 |
| PIKB/IKB | 1.00±0.10 | 13.11±1.34^***^ | 5.27±0.55^##^ | 322.431 | <0.05 |
| P-p65/p65 | 1.00±0.67 | 10.57±0.81^***^ | 5.07±0.25^##^ | 570.744 | <0.05 |

Note: HUVECs were treated with SAA1 or SAA1+TAK-242, with the untreated HUVECs as the control. The expression of MyD88, P-IKKα/β, IKKα/β, P-IKBα, IKBα, P-p65 and p65 was detected by Western blot. Data were analyzed using GraphPad Prism 8.3.0, the differences among multiple groups were determined by one-way ANOVA with Tukey’s post hoc test. ^*^means compared with the control group; ^#^means SAA1 compared with SAA1+TAK-242, n=6 (^***^*P<*0.001; ^#^*P<*0.05).

**Supplementary table 12 (corresponding to figure 8b).** Expression analysis of MyD88, P-IKKα/β, IKKα/β, P-IKBα, IKBα, P-p65 and p65 in macrophages derived from THP-1 cells following treatment with SAA1 or SAA1+TAK-242.

|  | Control | SAA1 | SAA1+TAK-242 | *F* | *P* |
| --- | --- | --- | --- | --- | --- |
| MyD88 | 1.00±0.11 | 3.89±0.45^***^ | 2.42±0.52^##^ | 76.990 | <0.05 |
| P-IKKα/β/IKKα/β | 1.00±0.10 | 10.87±0.95^***^ | 6.50±0.68^##^ | 321.001 | <0.05 |
| PIKB/IKB | 1.00±0.87 | 7.23±0.60^***^ | 4.96±0.17^##^ | 450.512 | <0.05 |
| P-p65/p65 | 1.00±0.19 | 3.67±0.21^***^ | 2.31±0.19^##^ | 411.102 | <0.05 |

Note: the macrophages derived from THP-1 cells induced by PMA were treated with SAA1 or SAA1+TAK-242, with the untreated macrophages as the control. The expression of MyD88, P-IKKα/β, IKKα/β, P-IKBα, IKBα, P-p65 and p65 was detected by Western blot. Data were analyzed using GraphPad Prism 8.3.0, the differences among multiple groups were determined by one-way ANOVA with Tukey’s post hoc test. ^*^means compared with the control group; ^#^means SAA1 compared with SAA1+TAK-242, n=6 (^***^*P<*0.001; ^##^*P<*0.05).
